# Could a hormone point the way to life extension?

**DOI:** 10.7554/eLife.00286

**Published:** 2012-10-15

**Authors:** Cynthia Kenyon

**Affiliations:** **Cynthia Kenyon** is at the University of California, San Francisco, United Statescynthia.kenyon@ucsf.edu

**Keywords:** longevity, fibroblast growth factor, growth hormone, liver, caloric restriction

## Abstract

Mice that have been genetically modified to produce high levels of fibroblast growth factor-21 live longer than mice with normal levels of this hormone.

**Related research article** Zhang Y, Xie Y, Berglund ED, Coate KC, He TT, Katafuchi T, Xiao G, Potthoff MJ, Wei W, Wan Y, Yu Rt, Evans RM, Kliewer SA, Mangelsdorf DJ. 2012. The starvation hormone, fibroblast growth factor-21, extends lifespan in mice. *eLife* **1**:e00065. doi: 10.7554/eLife.00065

**Image** Lifespans of normal mice (blue line) and mice overexpressing FGF-21 (red)

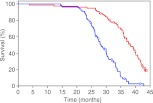

Will it be possible to slow the rate of human aging and extend lifespan? Maybe. Assuming that the first metazoans had short lifespans, gene changes have already extended our lifespans dramatically. Species with tissues much like ours can live far longer than we do, suggesting that perhaps we could too. In some human families, living healthily to age ninety or older is commonplace. With more knowledge of the genes responsible, it might be possible to design interventions to slow down aging and increase healthy lifespan.

In animals, many mutations can slow aging and extend lifespan, in some cases dramatically. Most of these life-extending mutations affect biochemical pathways that respond to nutrient, stress or energy levels. Inhibiting pathways that promote nutrient uptake or growth, or activating pathways that normally help the organism respond to stress or a shortage of energy, can extend lifespan in worms, flies and mice. Perhaps pharmaceutical techniques could be used to extend human lifespans by modifying these or other life-extension pathways.

Now, writing in *eLife*, Yuan Zhang of the University of Texas Southwestern (UTSW) Medical Center and co-workers report that a hormone, called fibroblast growth factor-21 (FGF-21), can extend lifespan in mice (Zhang et al., 2012). When food is withheld from an animal for 12 hours or longer, liver cells produce this hormone, which then mobilizes fat stores in the liver, and promotes the synthesis of glucose and ketones. FGF-21 also reduces basal insulin levels and increases insulin sensitivity. In addition, it suppresses further growth of the mice by preventing growth hormone from triggering the production of IGF-1 (insulin-like growth factor-1) in the liver.

Previously, a collaboration between the UTSW group and researchers at New York University School of Medicine engineered mice with levels of circulating FGF-21 that were 5–10 times higher than normal (Inagaki et al., 2008). As a consequence, these animals switched on their starvation response even though they continued to feed normally. Now Zhang et al. report that the median lifespans of these mice are increased—by 30% for male mice and 39% for female mice. One consequence of high levels of FGF-21, disruption of the growth hormone/insulin/IGF-1 pathway, is already known to extend the lifespan of mice by ∼50% (Coschigano et al., 2000). This disruption is likely to be responsible for the increase in the lifespans observed by Zhang et al. Other life extension pathways they examined (the TOR, AMPK and sirtuin pathways) all appeared normal in these mice.

Because FGF-21 is a hormone, it should be possible to increase its level in humans. It is possible that this will extend lifespan, because there are already hints that inhibiting the growth hormone/IGF-1 pathway promotes human longevity. First, genetic mutations that prevent cells from responding to IGF-1 are over-represented in centenarians (Suh et al., 2008), as are other rare DNA variants that reduce the activities of other components of this pathway, including the receptor for growth hormone (Y. Suh, personal communication). Second, DNA variants in the *FOXO3A* gene have been linked to exceptional longevity in at least eight studies across the world. FOXO proteins switch on genes that extend lifespan when insulin/IGF-1 signalling is inhibited in animals, although we do not know how these human DNA variants affect gene function. Third, genome-wide association studies for longevity highlight this pathway (Deelen et al., 2011).

It was the ability of FGF-21 to help animals survive starvation that initially prompted Zhang and co-workers – who are based at UTSW, the Howard Hughes Medical Institute and the Salk Institute – to test its role in aging. It has been known for decades that a reduced calorie intake (caloric restriction) can increase the lifespans of rodents and other animals. When first discovered, during the Great Depression of the 1930s, caloric restriction was assumed to work simply by reducing metabolic wear and tear. However, now we know that it extends lifespan by engaging specific signalling pathways and changing patterns of gene expression.

The results of a long-running caloric restriction experiment on primates were published recently and, unexpectedly, it was found that the calorically restricted monkeys did not live any longer than control animals (Mattison et al., 2012). This finding was puzzling because caloric restriction had seemed to extend lifespan in a previous study (Colman et al., 2009). However, the monkeys in the previous study had consumed a high-glycemic-index diet, and the control animals (the only group in either study with a relatively short lifespan) were allowed to eat all they wanted. The take-home message here might be that a healthy, moderate diet will provide just as much benefit as more severe food limitation. However, the differences between the outcomes of the two studies might be partly due to other factors, such as genotypic differences between the groups. Different strains of mice are now known to respond to caloric restriction in different ways: some strains even live shorter, not longer.

While caloric restriction is not fully understood, Zhang et al. show that it is unlikely to explain their results because caloric restriction does not trigger FGF-21 production. Intriguingly, however, recurrent periods of starvation, administered in the form of intermittent, every-other-day feeding, can also extend lifespan in animals. This regime does not reduce overall caloric intake, as the animals overeat when food is available, but it should trigger FGF-21 production. Moreover, in *C. elegans*, where the mechanism has been examined, the life extension produced by intermittent fasting appears to act by inhibiting insulin/IGF-1 signalling and activating FOXO (Honjoh et al., 2009). It would be interesting to know whether every-other-day feeding might extend the lifespans of primates.

One of the most exciting features of many long-lived worms, flies and mice is their resistance to age-related diseases such as cancer, atherosclerosis and protein-aggregation disease. Since FGF-21 activates the same longevity pathways, it is possible that this hormone might also confer resistance against disease. Unfortunately, like some other mice that are deficient in IGF-1, these long-lived mice have low bone density, so we cannot recommend injecting FGF-21 right now.
